# Direct observation of redox reactions in *Candida parapsilosis* ATCC 7330 by Confocal microscopic studies

**DOI:** 10.1038/srep34344

**Published:** 2016-10-14

**Authors:** Sowmyalakshmi Venkataraman, Shoba Narayan, Anju Chadha

**Affiliations:** 1 Laboratory of Bioorganic Chemistry, Department of Biotechnology, Indian Institute of Technology Madras, Chennai 600 036, India; 2 National Center for Catalysis Research, Indian Institute of Technology Madras, Chennai 600 036, India

## Abstract

Confocal microscopic studies with the resting cells of yeast, *Candida parapsilosis* ATCC 7330, a reportedly versatile biocatalyst for redox enzyme mediated preparation of optically pure secondary alcohols in high optical purities [enantiomeric excess (ee) up to >99%] and yields, revealed that the yeast cells had large vacuoles under the experimental conditions studied where the redox reaction takes place. A novel fluorescence method was developed using 1-(6-methoxynaphthalen-2-yl)ethanol to track the site of biotransformation within the cells. This alcohol, itself non-fluorescent, gets oxidized to produce a fluorescent ketone, 1-(6-methoxynaphthalen-2-yl)ethanone. Kinetic studies showed that the reaction occurs spontaneously and the products get released out of the cells in less time [5 mins]. The biotransformation was validated using HPLC.

*Candida parapsilosis* ATCC 7330 is an established biocatalyst for the preparation of numerous optically pure aryl/ aliphatic secondary alcohols and aryl amines using different strategies namely, asymmetric reduction^1^,^2^,^3^, deracemisation^4^,^5^,^6^ and oxidative kinetic resolution^7^. These optically pure secondary alcohols are produced from either prochiral ketones or racemic alcohols in reaction times as short as 5 min or even up to 24 h. These small organic molecules which are biotransformed as reported in this study, possibly enter the cells by passive diffusion i.e. transport of molecules across the membranes from higher to lower concentration without any additional energy requirement^8^. Once inside the cell, the molecule gets transformed and is excreted from the cell due to which it is possible to extract the material from the reaction mixture and determine its optical purity, spectral analysis and yield. The present study addresses the question about the site of biotransformation inside the cell. A fluorogenic assay for confocal microscopic studies was developed for this purpose. The experimental results were validated using HPLC.

The most widely used methods for localisation studies are subcellular fractionation, immunohistochemical studies and fluoro-probe techniques. It is known that cells consist of different dehydrogenases and other metabolic enzymes even under extremely unfavourable conditions^9^. In the case of the three reported bacterial dehydrogenases, class I ADHs, are localised in the periplasm; class II ADHs are present as soluble monomers in the periplasm and class III ADHs are present in acetic acid bacteria and are also localised in the periplasmic side of the cytoplasmic membrane^10^. Localisation of alcohol dehydrogenases (ADHs) in various mammalian tissues mainly use immunohistochemical studies^11^,^12^, immuno-gold labeling^13^, Western blotting^14^ and fluorimetric assays^15^. Only a few reports are available till date on the localisation of alcohol dehydrogenases in yeast cells. ADH-I and ADH-II were localised in the cytosol while ADH-III was localised in the mitochondrial matrix based on subcellular fractionation^16^,^17^. Using a green fluorescent protein, a commonly used fluorescent tag, obtained from the jelly fish *Aequorea victoria*, ADH-IV and ADH-V were localised in the mitochondria and cytoplasm respectively^18^; and the localisation of ADH VI and VII which were obtained from over-expression studies remains ambiguous^18^,^19^,^20^. However, tracking the site of biotransformation of small organic molecules (alcohols/ketones) by resting yeast cells is not yet reported.

Klein *et al*., developed an enantioselective fluorimetric assay for alcohol dehydrogenases based on umbelliferone. The chiral secondary alcohol is tagged with umbelliferone. On oxidation in the presence of ADH, the corresponding unstable ketone formed undergoes β-elimination at basic pH in the presence of bovine serum albumin to release the fluorescent umbelliferone^21^. This strategy can be used when the target protein is a single enzyme. In whole cells where a pool of different enzymes is present, multiple reactions can take place which can be misleading. Ideally, the substrate molecule should be detectable without a tag, as the fluorescent tag can alter its properties including that of permeation through membranes. In many studies, the total ADH activity is determined spectrophotometrically using *p*-nitroso-N,N-dimethyl aniline to the fluorescent *p*-amino-N,N-dimethyl aniline^22^. The most commonly used fluorogenic substrates for ADH activity are 4-methoxy-1-naphthaldehyde and 6-methoxy-2-naphthaldehyde, which are more used to determine ADH-1 and ADH-2 activities respectively in mammalian cells^23^,^24^,^25^. Unlike mammalian cells, yeasts have thick cell wall, which has to factored in while considering the entry of bulky fluorescent substrates in and out of the cell.

This paper presents for the first time, experiments using fluorescent organic small molecules to track their biotransformation [redox reaction] in the yeast cells. The biotransformed product then comes out of the cells.

## Results

### Selection of the substrate

**Figure 1:**
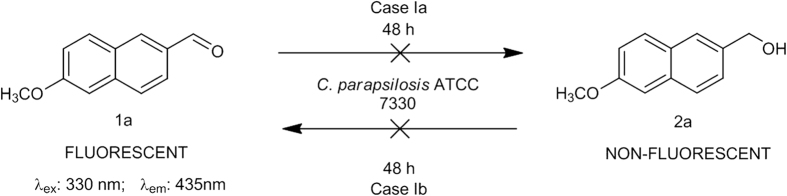
Biocatalytic reduction of 6-methoxy-2-naphthaldehyde 1a (Case Ia) and oxidation of (6-methoxynaphthalen-2-yl)methanol 2a using *C. parapsilosis* ATCC 7330 (Case Ib).

The fluorescent substrate, 6-methoxy-2-naphthaldehyde **1a** (Fig. 1) is often used to study the ADH activity for different mammalian cells^23^,^24^,^25^. This molecule was incubated with wet cells of *C. parapsilosis* ATCC 7330 using ethanol as the cosolvent and glucose as the cosubstrate^1^. Two possibilities were considered: a. If the substrate aldehyde **1a** got reduced by *C. parapsilosis* ATCC 7330, it would give the product alcohol which is not fluorescent i.e, (6-methoxynaphthalen-2-yl)methanol **2a** (**Case Ia**) and b. If the alcohol **2a** was used as substrate which then gets oxidised to the aldehyde **1a** by *C. parapsilosis* ATCC 7330, it would mean going from a non-fluorescent molecule to a fluorescent molecule (**Case Ib**), which is preferred for tracking. The reaction was initially carried out for 24 h and later extended to 48 h, and the product formation was monitored by TLC. The TLC indicated no trace of product **2a** formation even after 48 h. The next experiment was carried out (**Case Ib;** Fig. 1) where the alcohol **2a** was the substrate for biocatalytic oxidation (Tris-HCl buffer, pH 8.5, 10 mM) with acetone as cosubstrate and ethanol as cosolvent^26^. The progress of the reaction as monitored by TLC did not show aldehyde formation and the substrate remained unreacted even after 48 h.

**Figure 2:**
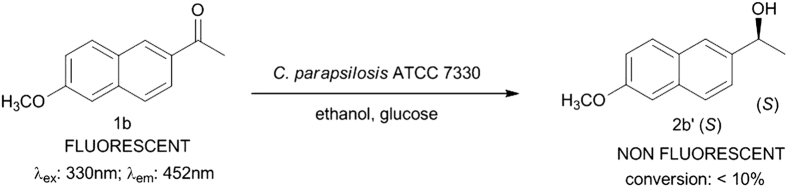
Asymmetric reduction of 1-(6-Methoxynaphthalen-2-yl)ethanone 1b using *C. parapsilosis* ATCC 7330 (Case IIa).

**Figure 3:**
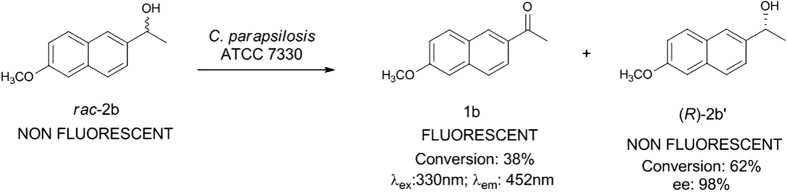
Enantioselective oxidation of *rac*-1-(6-Methoxynaphthalen-2-yl)ethanol 2b using *C. parapsilosis* ATCC 7330 (Case IIb).

Earlier reports from our lab have shown that *C. parapsilosis* ATCC 7330 mediated the asymmetric reduction^1^,^2^,^3^,^27^ and deracemisation^4^,^28^,^29^,^30^ of aromatic/aliphatic prochiral ketones and secondary alcohols respectively. It seemed logical to use a prochiral ketone for the biocatalytic reduction which shows structural similarity to **1a** for the study. Hence, 1-(6-methoxynaphthalen-2-yl)ethanone **1b**, a fluorogenic aldol sensor in antibody-catalysed retro-aldol reaction^31^, was chosen as the next substrate (**Case IIa;** Fig. 2) and on incubation with cells of *C. parapsilosis* ATCC 7330 under reaction conditions optimised earlier, showed only a trace of non-fluorescent product alcohol [1-(6-methoxynaphthalen-2-yl)ethanol] **2b′** by TLC. The conversion was only <10% as monitored by HPLC using a reverse phase column. This substrate **1b** could not be used for further studies and in any case the product alcohol is non-fluorescent. The racemic alcohol **2b** (**Case IIb**) was used as a substrate (substrate concentration: 2.8 mM) for deracemisation using the earlier optimised conditions^26^ and the reaction was carried out for 1 h. The reaction was expected to proceed *via* stereoinversion mechanism like other aryl racemic alcohols carried out using this biocatalyst^4^,^5^,^6^. The progress of the reaction was monitored using TLC and the intermediate formation of the fluorescent ketone **1b** was observed along with the alcohol. The time course of the reaction was monitored by HPLC using the reverse phase column and observed that 38% of ketone **1b** along with the unreacted alcohol **2b′** were formed (Supplementary Results, Supplementary Fig. 1), which indicated that the reaction which occurred was oxidative kinetic resolution (Fig. 3). The same reaction when carried out in the absence of acetone, gave only 27% of ketone which showed the important role of acetone in cofactor regeneration^32^. The chiral HPLC profile showed that the (*S*)-enantiomer of the racemate undergoes enantioselective oxidation, while the (*R*)-enantiomer (ee 98%) remains unreacted (Fig. 3). The time course of this reaction from the chiral HPLC profile showed that the reaction is completed within 30 minutes and further extending the reaction time up to 1 h did not show any noticeable change in the conversion of the products formed (Supplementary Fig. 2).

### Substrate concentration

Substrate concentrations in the range 0.0162–3.38 mM were studied using constant biomass (1.2 g wet cells/5 mL). The concentration of the substrate **2b** was optimised at 0.87 mM for maximum conversion (38%) to **1b** (Supplementary Fig. 3) and used for all further experiments.

### Effect of culture age on alcohol dehydrogenase activity of the biocatalyst

All the above experiments were carried out with the whole cells harvested at 14^th^ hour (mid log phase) of the growth phase^1^. In order to understand the effect of enzyme activity with respect to the culture age, the cells were harvested at different time points (8–40 h) and used for the biotransformation. The results showed that cells harvested at the 24^th^ hour of growth, showed the maximum conversion [49%] to ketone (Supplementary Fig. 4). Even though cells harvested at 14 h produced the same ketone at less conversion (38%), these cells were used to visualise the site of biotransformation. For several other substrates reported earlier from our lab, the 14 h harvested cells produced the desired optically pure products with maximum ee (up to >99%)^1^,^2^,^3^,^6^,^7^,^26^,^29^,^30^,^33^,^34^,^35^.

### Cell imaging studies using confocal microscopy

**Figure 4:**
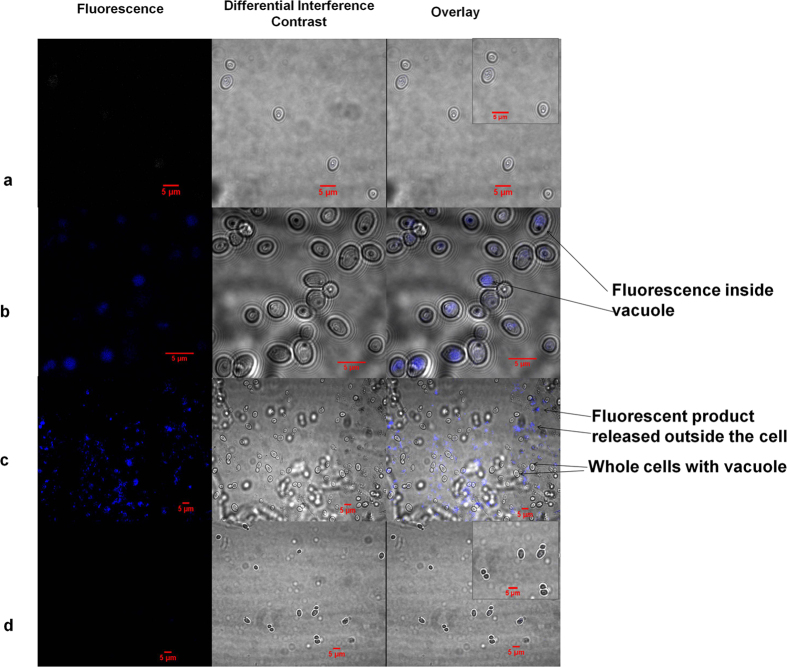
Microscopic images of localisation of biotransformation in *C. parapsilosis* ATCC 7330 in pH 8 sodium phosphate buffer using Confocal laser microscopy (**a**) Cells with no substrate added; inset: enlarged cells without substrate (**b**) Cells incubated with *rac*-1-(6-Methoxynaphthalen-2-yl)ethanol 2b with appearance of fluorescence inside the vacuole; (**c**) Release of the fluorescent product 1-(6-Methoxynaphthalen-2-yl)ethanone 1b outside the cell; (**d**) Heat killed cells incubated with *rac*-1-(6-Methoxynaphthalen-2-yl)ethanol 2b with no fluorescent product formation; inset: enlarged heat killed cells with substrate.

The reaction was carried out qualitatively on a microscope slide using a higher substrate concentration (4.4 mM) and added over the cell suspension as a mixture in acetone: ethanol (2:1). The substrate concentration was increased in anticipation that the distribution of the substrate to the cells may not be uniform due to the absence of shaking during the reaction and also the optimised concentration (0.87 mM) might be too low to be detected if the distribution is not uniform. From the images, obtained with the live cells, it was observed that the fluorescence was localised in the vacuole of the cell. Another, interesting observation made from the images is that the formation of the fluorescent ketone molecule occurs in the vacuole region of the cell and as soon as the ketone is formed, it gets released out of the cell in less than 5 min (Fig. 4). The control experiments (using heat killed cells) did not show the formation of ketone (absence of fluorescence within the cells). This confirms the formation of the fluorescent molecule in the live cells due to the active alcohol dehydrogenase enzyme present.

### Rate of formation of the fluorescent ketone

The images show that the formation of ketone **1b** from the racemate **2b** occurs inside the vacuole and gets released from the cell spontaneously. The kinetics of ketone formation was followed under optimised reaction conditions. Aliquots at time 0 to 5 min were analysed using reverse phase HPLC and the amount of ketone present in the supernatant was estimated using a standard plot of known concentrations (0.05–0.4 mM) of ketone **1b.** It was observed that the formation of the fluorescent ketone begins in the 1^st^ minute (at the rate of 136.67 μg/min/gm of biomass) and the maximum conversion to the fluorescent ketone (up to 38%, 0.32 mM) is achieved in the 3^rd^ minute of the reaction time for this substrate concentration (0.87 mM) (Supplementary Fig. 5). The important observation from this study is that the formation of ketone occurs spontaneously and gets released out of the cells, possibly making way for the other set of non-fluorescent alcohol molecules to get converted to the ketone.

## Discussion

The oxidative kinetic resolution of racemic 1-(6-methoxynaphthalen-2-yl)ethanol **2b** by *Candida parapsilosis* ATCC 7330 is a good assay to track the biotransformation in the yeast cells as shown in this study.

Designing fluorogenic substrates which are capable of coupling with the chemical redox reaction and exhibiting the switch in emission properties is reported^36^. Most of the organic fluorophores designed as redox probes are based on the push-pull structural feature, whereby the electron donating and electron withdrawing groups are connected *via* an extended π-conjugated system^37^. The carbonyl group becomes part of the push-pull system and when it gets reduced to the alcohol, the electron withdrawing nature of the carbonyl group changes to the electron donating nature of the hydroxyl group. This shift causes changes in the electronic properties between the reactant and product giving different emission characteristics^36^. Initially, the substrate 6-methoxy-2-naphthaldehyde **1a** was chosen for this study as it satisfies the conditions of an ideal organic fluorescent redox probe. However, substrate **1a** remained as such up to 48 h. Likewise, alcohol **2a** was not oxidised to the fluorescent aldehyde as expected. Both **1a** and **2a** are not preferred substrates for this biocatalyst, although the asymmetric reduction of α-oxoaldehydes using this biocatalyst is known to produce the respective diols^2^. In the case of oxidation, primary alcohols are less preferred as compared to secondary alcohols^7^. From the earlier reports^1^,^3^,^27^ the asymmetric reduction of prochiral ketones using *C. parapsilosis* ATCC 7330 is well documented. Therefore, the substrate **1b** was subjected to asymmetric reduction but it resulted in low conversion (<10%) to the product alcohol, a phenomenon seen for substrates with bulky groups including naphthyl group which show either less or no conversion to the product alcohols^4^,^38^,^39^.

Most yeast cells including *C. parapsilosis* ATCC 7330 appear as ovoid or ellipsoidal in shape with a cell length of 2–3 μm to 20–50 μm. As expected, the size of the yeast cell differs with its age and growth conditions^40^. During unfavourable conditions e.g. nutritional deprivation or oxidative stress, the cytoplasmic proteins and the cell organelles get transported to a vacuole by means of non-selective “cytoplasm-to-vacuole” pathway which tends to overlap with the autophagy for degradation^9^. It is reported that several NAD^+^ dependent alcohol dehydrogenases are found in the vacuolar compartment of *S*. *cerevisiae*^41^,^42^. Evidently, vacuoles of yeast cells undergo morphological changes in response to various intracellular and extracellular stimuli. In the log phase, cells consist of multiple medium-sized vacuoles under normal conditions. These multiple vacuoles, fuse into a single large vacuole during the stationary phase or under nutritional deprivation. They can also get fragmented into multiple small vesicles during osmotic stress. In hypo-osmotic conditions, the vacuoles swell into a single large vacuole occupying a majority of the cellular volume. Thus, the uptake and release of water or ions from the vacuole shows a distinct morphological change in the size and number of vacuoles in the yeast cell^43^.

In the present study, *C. parapsilosis* ATCC 7330 cells were harvested from the growth medium at 14 h, which corresponds to the mid log phase of the growth period. All the experiments were carried out using the harvested cells or resting cells. Due to nutritional stress under these conditions, the cellular contents including the cytoplasmic proteins are forced to get transported into the vacuole^9^ inside the yeast cells. Therefore, under these experimental conditions the representative enantioselective oxidation of *racemic*-1-(6-methoxynaphthalen-2-yl)ethanol to the fluorescent 1-(6-methoxynaphthalen-2-yl)ethanone occurs in the vacuoles inside the cells (Fig. 4).

A novel fluorescence based method for the site of localisation of the oxidation reaction of 1-(6-methoxynaphthalen-2-yl)ethanol to the fluorescent 1-(6-methoxynaphthalen-2-yl)ethanone in *C. parapsilosis* ATCC 7330 in addition to reporting a biocatalysed method to prepare the optically pure (*R)*-1-(6-methoxynaphthalen-2-yl)ethanol and the precursor ketone 1-(6-methoxynaphthalen-2-yl)ethanone is reported in this study. The experimental results show that the enzymes like alcohol dehydrogenases which are used to catalyse various redox reactions are localised in the vacuole of the yeast cells under the conditions studied. *Racemic*-1-(6-methoxynaphthalen-2-yl)ethanol was oxidised using *C. parapsilosis* ATCC 7330 enantioselectively to the fluorescent ketone (38%), leaving the (*R*)-enantiomer unreacted with 98% ee and 62% conversion. The study highlights that the fluorescent ketone once formed in the vacuole gets released out of the cell in less time, which was confirmed from the kinetic studies.

## Methods

The chemicals, 6-methoxy-2-naphthaldehyde and 1-(6-methoxynaphthalen-2-yl)ethanone were purchased from Sigma-Aldrich and standard racemic alcohols were synthesised using the reported method^44^. The growth conditions of *Candida parapsilosis* ATCC 7330 were followed as mentioned earlier^1^. The fluorescent intensity of the substrates and products were determined using JASCO spectrofluorimeter FP 8000. The conversion and ee of the products formed were analysed using JASCO PU-1580 HPLC equipped with PDA detector. The confocal microscopic studies were carried out using Zeiss LSM 700 Confocal microscopy and image processing was done using Zen LE- 2012 software.

### Enantioselective oxidation of 1-(6-methoxynaphthalen-2-yl)ethanol 1b using *Candida parapsilosis* ATCC 7330

In a 25 mL Erlenmeyer flask, 1.2 g of wet cells of *Candida parapsilosis* ATCC 7330 was suspended in 5 mL of sodium phophate buffer (pH 8, 10 mM). The substrate 1-(6-methoxynaphthalen-2-yl)ethanol **1b** (2.8 mM) dissolved in 100 μL of ethanol and 200 μL of acetone, was added and the reaction was continued up to 30 min. The progress of the reaction was monitored using TLC and after the reaction time the products were extracted using ethyl acetate. The conversion was determined using HPLC with MERCK Lichrospher 100 RP-18e column with acetonitrile: water (85:15) as mobile phase (flow rate 0.5 mL/min) and ee using Chiralcel OD-H column (hexane: 2-propanol; 95:5; flow rate: 1 mL/min)^45^ respectively. All the experiments were carried out in triplicate and the control experiments were done in parallel using blank (no cells) as well as using heat-killed cells under identical conditions.

### Cell imaging studies using confocal microscopy

The experiments were carried out on microscopic slides by adding 20 μL of the cell suspension (OD_600_ 0.7; pH 8 buffer). The number of cells of *C. parapsilosis* ATCC 7330 present in the cell suspension was found to be 6.2 × 10^5^ cells/20 μL which was counted using haemocytometer^46^. About 10 μL of substrate **2b** (4.4 mM) in acetone:ethanol mixture (2:1) was added to the cell suspension in the slide. The slide was mounted on the confocal microscope and images were captured using the constant instrumental settings (Supplementary Table 1). The experiments were carried out in triplicate and the control experiments were done in parallel using blank cells (without substrate) as well as using heat-killed cells under identical conditions.

### Reaction kinetics of the formation of 1-(6-methoxynaphthalen-2-yl)ethanone 1b from racemic 1-(6-methoxynaphthalen-2-yl)ethanol 2b using *Candida parapsilosis* ATCC 7330

In a 25 mL Erlenmeyer flask, 1.2 g of wet cells of *Candida parapsilosis* ATCC 7330 was suspended in 5 mL of sodium phophate buffer (pH 8, 10 mM). The substrate 1-(6-methoxynaphthalen-2-yl)ethanol **2b** (0.87 mM) dissolved in 100 μL of ethanol and 200 μL of acetone, was added and the reaction was continued up to 5 min. The aliquots were taken at 1 min intervals and the products were extracted using ethyl acetate. The conversion was determined using HPLC using the conditions mentioned above.

A series of known concentrations (0.05–0.4 mM) of 1-(6-methoxynaphthalen-2-yl)ethanone **1b** were suspended in 5 mL of sodium phophate buffer (pH 8, 10 mM) and were extracted using ethyl acetate. The area under the curve was determined using HPLC using the conditions mentioned above. The standard plot was drawn earlier to correlate the area under the curve with respect to the concentration of the ketone **1b**.

### Preparative scale synthesis of 1-(6-methoxynaphthalen-2-yl)ethanone 1b by enantioselective oxidation of racemic-1-(6-methoxynaphthalen-2-yl)ethanol 2b using the whole cells of *Candida* parapsilosis ATCC 7330

In a 150 mL Erlenmeyer flask, 15 g of wet cells of *Candida parapsilosis* ATCC 7330 was suspended in 28.2 mL of sodium phosphate buffer (pH 8, 10 mM), 1.2 mL of acetone was added to it and incubated at 25 °C and 150 rpm. The substrate, 1-(6-methoxynaphthalen-2-yl)ethanol **2b** (80 mg, 0.4 mmol) which was dissolved in 600 μL of ethanol was added and the reaction was continued for 30 min. After the reaction time, the crude product was extracted thrice with ethyl acetate, the organic layer was dried over anhydrous sodium sulphate and the solvent was removed by rotary evaporator. The optically pure (*R*)-1-(6-methoxynaphthalen-2-yl)ethanol **2b′** (ee: 98%, yield: 47%) and 1-(6-methoxynaphthalen-2-yl)ethanone **1b** (yield: 41%) were obtained as colourless solids after purification with silica gel column chromatography using hexane:ethyl acetate (90:10) as eluent. The purified products were characterised using spectroscopic (IR, ^1^H and ^13^C NMR) techniques and were consistent with the literature reported values^45^,^47^ (Supplementary Figs 6 to 9).

### Spectral Characterisation of biotransformed products. 1-(6-Methoxynaphthalen-2-yl)ethanone 1b


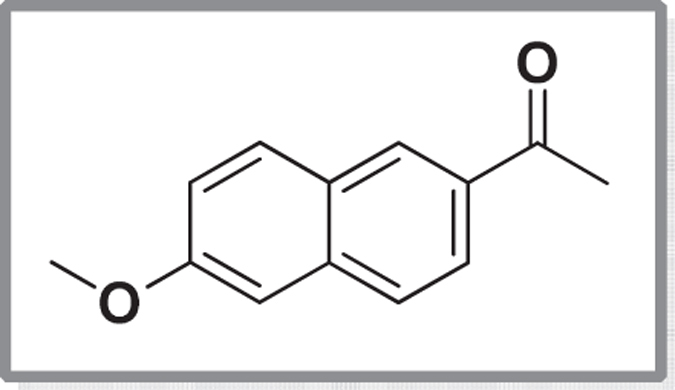
Colourless solid, ^1^H NMR (500 MHz; CDCl_3_; δ in ppm)^47^: 8.38 (d, *J* *=* 1 Hz, 1H), 7.99 (dd, *J* *=* 8.5, 2 Hz, 1H), 7.83 (d, *J* *=* 9 Hz, 1H), 7.75 (d, *J* *=* 9 Hz, 1H), 7.19 (dd, *J* *=* 9, 2.5 Hz, 1H), 7.14 (d, *J* *=* 2 Hz, 1H), 3.93 (s, 3H), 2.68 (s, 3H); ^13^C NMR (CDCl_3_; 125 MHz; δ in ppm): 197.83, 159.71, 137.23, 132.56, 131.06, 130.01, 127.76, 127.04, 124.61, 119.67, 105.70, 55.37, 26.50; IR (neat; cm^−1^): 3060, 3000, 2969, 2938, 1733.

### (*R*)- 1-(6-Methoxynaphthalen-2-yl)ethanol 2b'


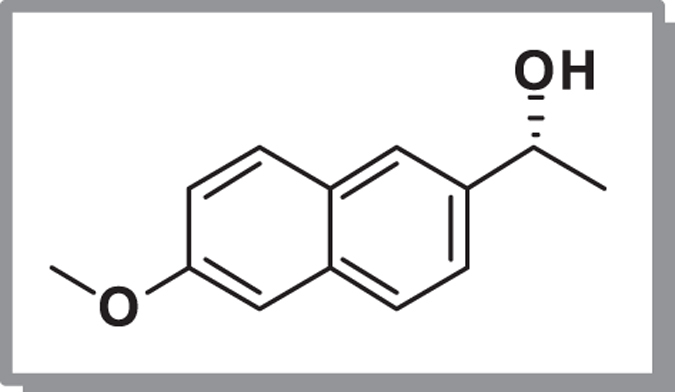
Colourless solid, ^1^H NMR (500 MHz; CDCl_3_; δ in ppm): 7.71–7.74 (m, 3H), 7.47 (dd, *J* *=* 8.5, 1.5 Hz, 1H), 7.12–7.16 (m, 2H), 5.04 (q, *J* *=* 6.5 Hz, 1H), 3.92 (s, 3H), 1.57 (d, *J* *=* 6.5 Hz, 3H); ^13^C NMR (CDCl_3_; 125 MHz; δ in ppm): 157.65, 140.89, 134.04, 129.39, 128.73, 127.16, 124.35, 123.76, 118.95, 105.68, 70.53, 55.30, 25.05; IR (cm^−1^): 3343, 3008, 2966, 2935, 1074; Specific rotation: [α]_D_^26^ + 28.4 (c 0.8, CHCl_3_). The compound was resolved by HPLC using the Chiralcel OD-H column using hexane: 2-propanol (95:5) as mobile phase with flow rate 1.0 mL/min with retention times (min): 14.92 (*S*, minor); 21.13 (*R*, major)^45^.

## Additional Information

**How to cite this article**: Venkataraman, S. *et al*. Direct observation of redox reactions in *Candida parapsilosis* ATCC 7330 by Confocal microscopic studies. *Sci. Rep*. **6**, 34344; doi: 10.1038/srep34344 (2016).

## Supplementary Material

Supplementary Information
