# Supplementary material for: Direct observation of redox reactions in Candida parapsilosis ATCC 7330 by Confocal microscopic studies
Source: Sci Rep. 2016 Oct 14;6:34344. doi: 10.1038/srep34344 (PMC5064409; doi:10.1038/srep34344)
Supplement: Supplementary Information [file srep34344-s1.pdf]

Supplementary Results

Direct observation of redox reactions in *Candida parapsilosis* ATCC 7330 by Confocal microscopic studies

Sowmyalakshmi Venkataraman,[a,c] Shoba Narayan[a,d] and Anju Chadha[a,b*]

a. Laboratory of Bioorganic Chemistry, Department of Biotechnology,

b. National Center for Catalysis Research,

Indian Institute of Technology Madras, Chennai 600 036, India

Tel.: +91 44 2257 4106; Fax: +91 44 2257 4102, E-mail: anjuc@iitm.ac.in

Present address: [c] Department of Pharmaceutical Chemistry, School of Pharmaceutical Sciences, Vels University (VISTAS), Chennai 600 117, India.

[d] Faculty of Allied Health Sciences, Chettinad Academy of Research and Education, Kelambakkam, Chennai 603 103, India

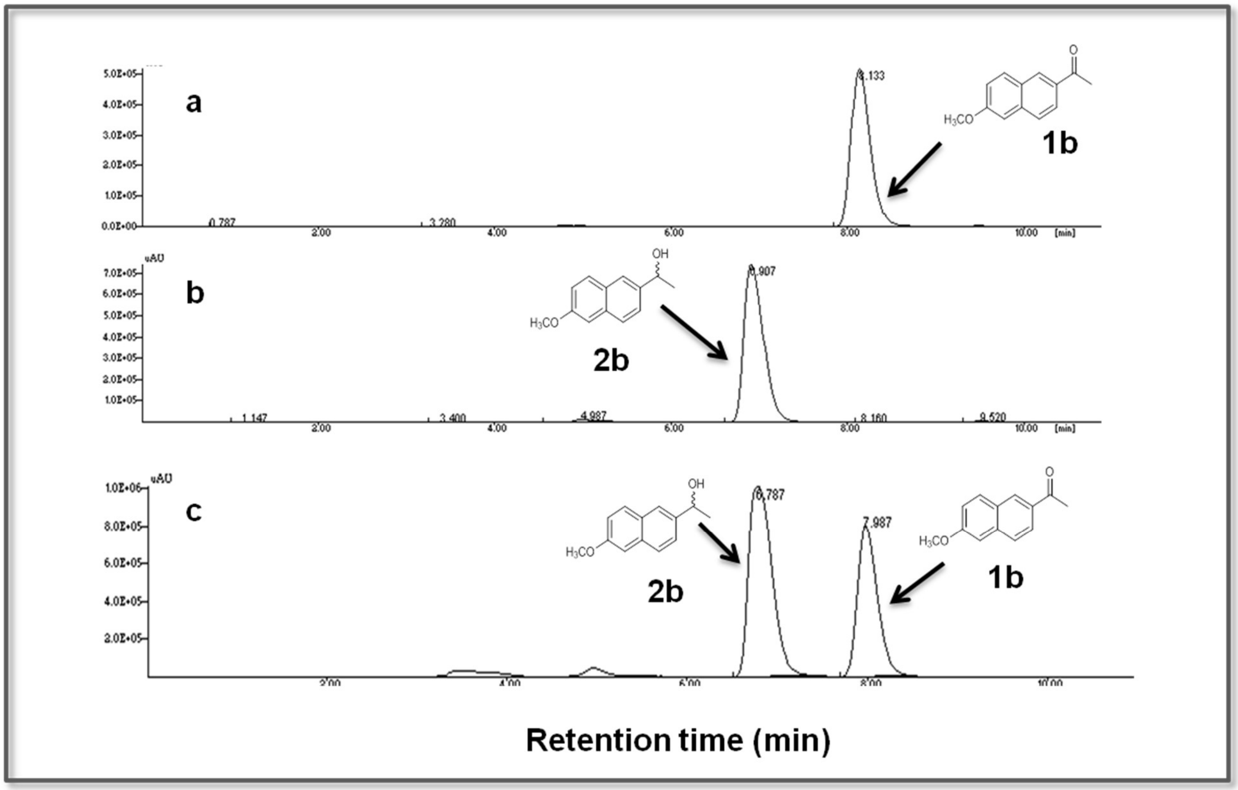

Supplementary Figure 1: Reverse phase HPLC profile of the biotransformation of *rac*-1-(6-methoxynaphthalen-2-yl)ethanol 2b using *C. parapsilosis* ATCC 7330. **a**) Standard 1-(6-methoxynaphthalen-2-yl)ethanone **1b; b)** *Racemic* 1-(6-methoxy naphthalen-2-yl)ethanol **2b**; **c)** Enantioselective oxidation of *rac*-1-(6-methoxy naphthalen-2-yl)ethanol **2b** showing 1-(6-methoxynaphthalen-2-yl)ethanone **1b** and 1-(6-methoxynaphthalen-2-yl)ethanol **2b**

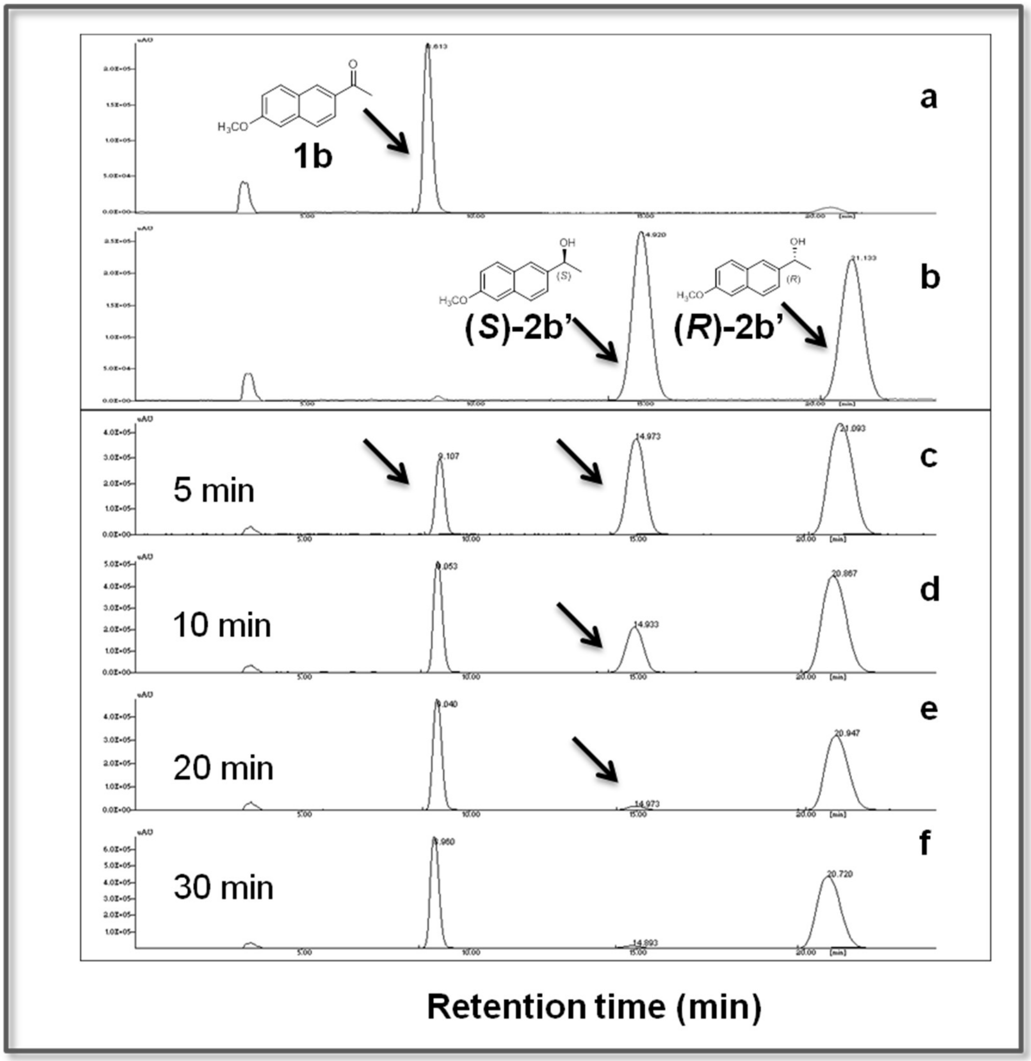

Supplementary Figure 2: Chiral HPLC profile of the biotransformation of *rac*-2b using *C. parapsilosis* ATCC 7330. **a)** Standard 1-(6-methoxynaphthalen-2-yl)ethanone **1b; b)** *Racemic* 1-(6-methoxynaphthalen-2-yl)ethanol **2b**; **c-f**) Time course of enantioselective oxidation of *rac*-1-(6-methoxynaphthalen-2-yl)ethanol **2b**

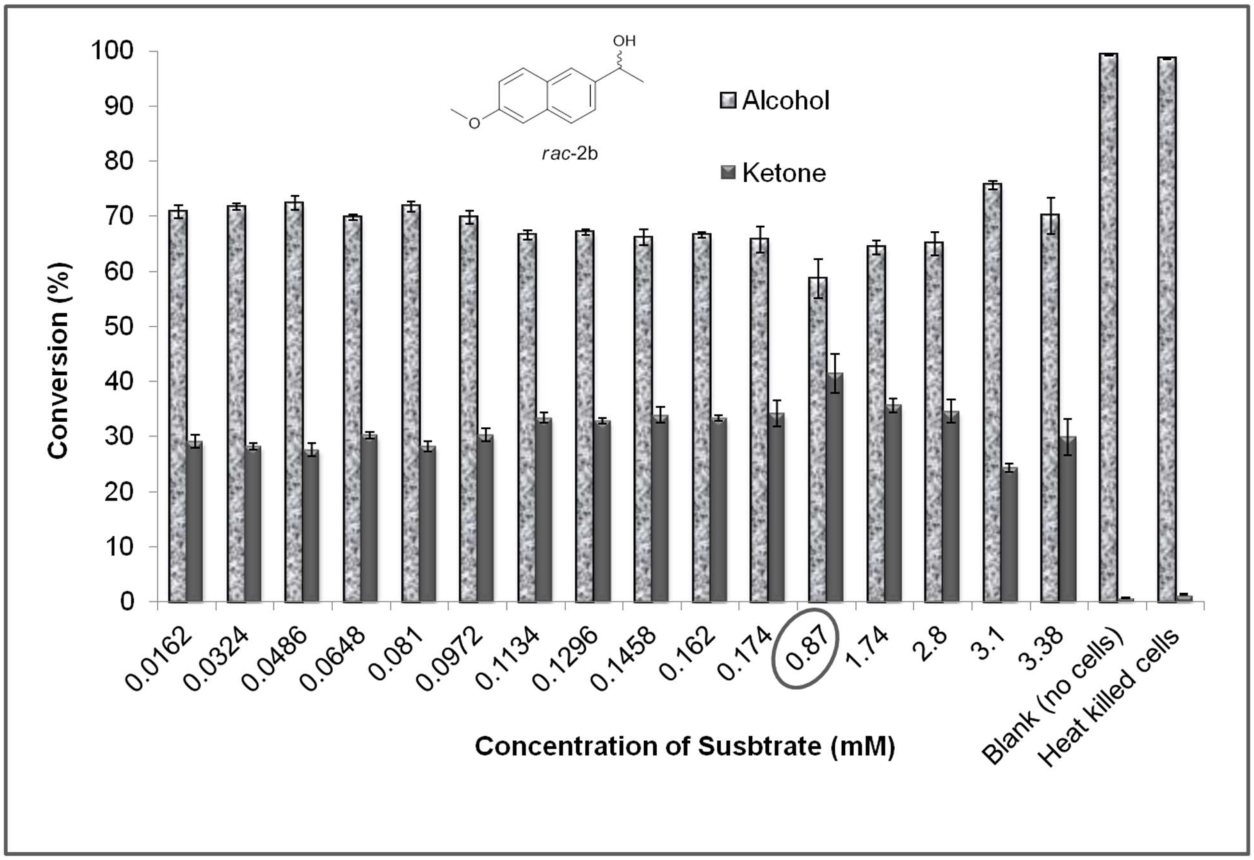

Supplementary Figure 3: Optimisation of substrate concentration for the biotransformation of non-fluorescent alcohol *rac*-1-(6-methoxynaphthalen-2-yl)ethanol **2b** to fluorescent ketone 1-(6-methoxynaphthalen-2-yl)ethanone **1b;** each experiment was done in triplicate and the % conversion values of ketone and alcohol shown are mean values with standard deviations

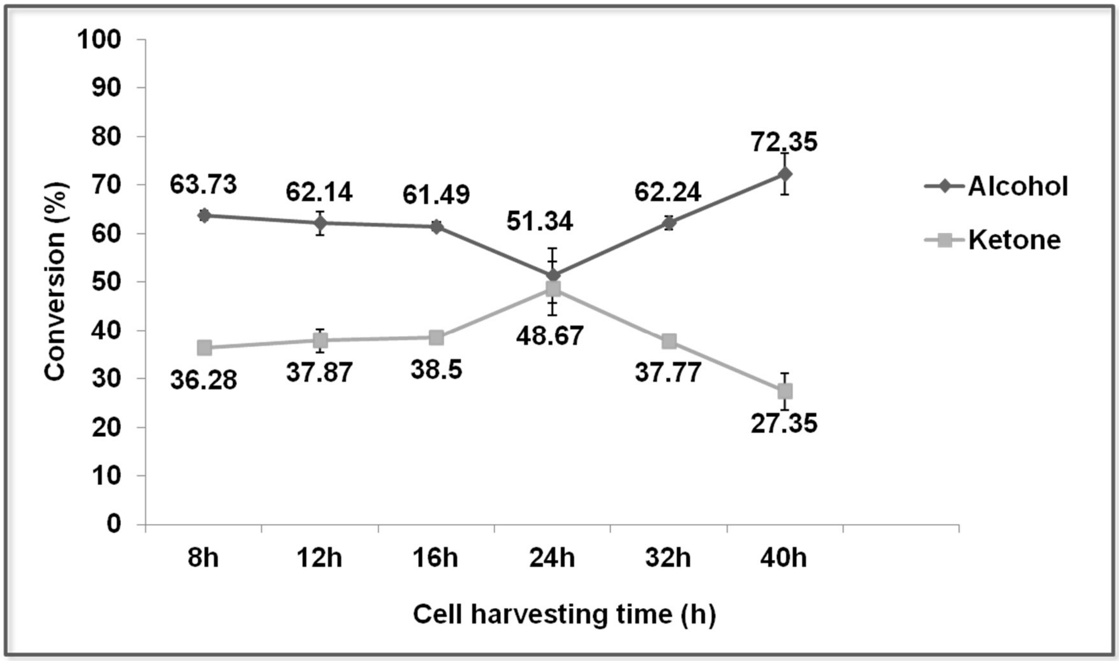

Supplementary Figure 4: Effect of culture harvesting time on alcohol dehydrogenase activity of *C. parapsilosis* ATCC 7330; each experiment was done in triplicate and the % conversion values of ketone and alcohol shown are mean values with standard deviations

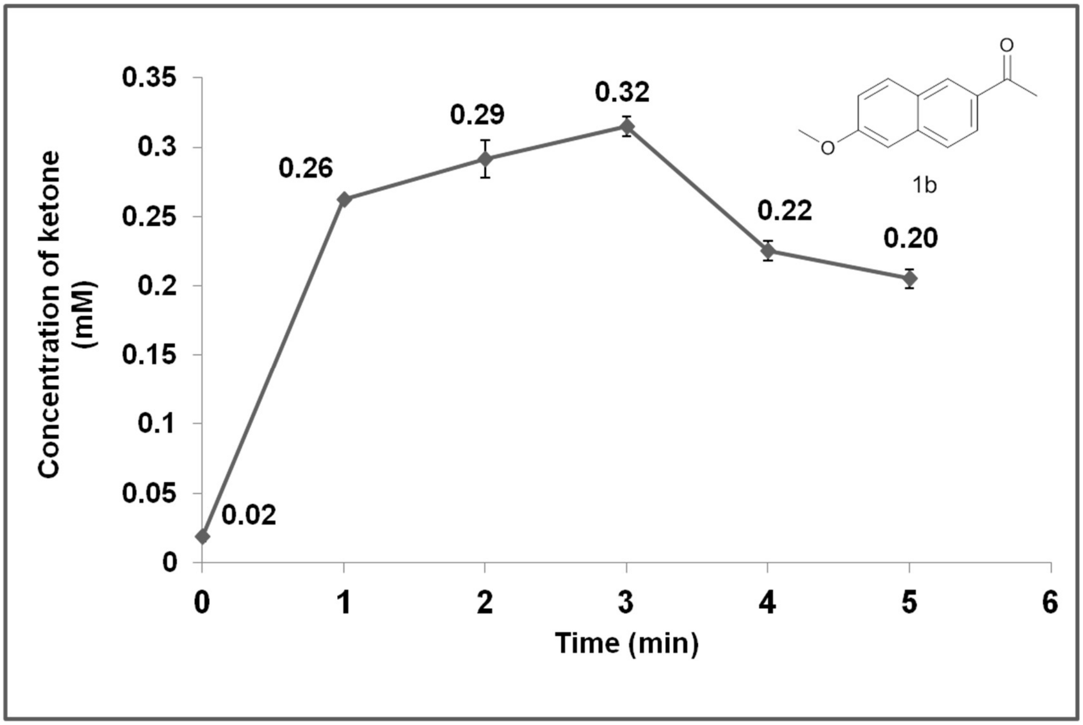

Supplementary Figure 5: Concentration of fluorescent ketone 1-(6-methoxy naphthalen-2-yl)ethanone **1b** from *racemic* 1-(6-methoxynaphthalen-2-yl)ethanol **2b** using *C. parapsilosis* ATCC 7330; each experiment was done in triplicate and the concentration of ketone formed are mean values with standard deviations

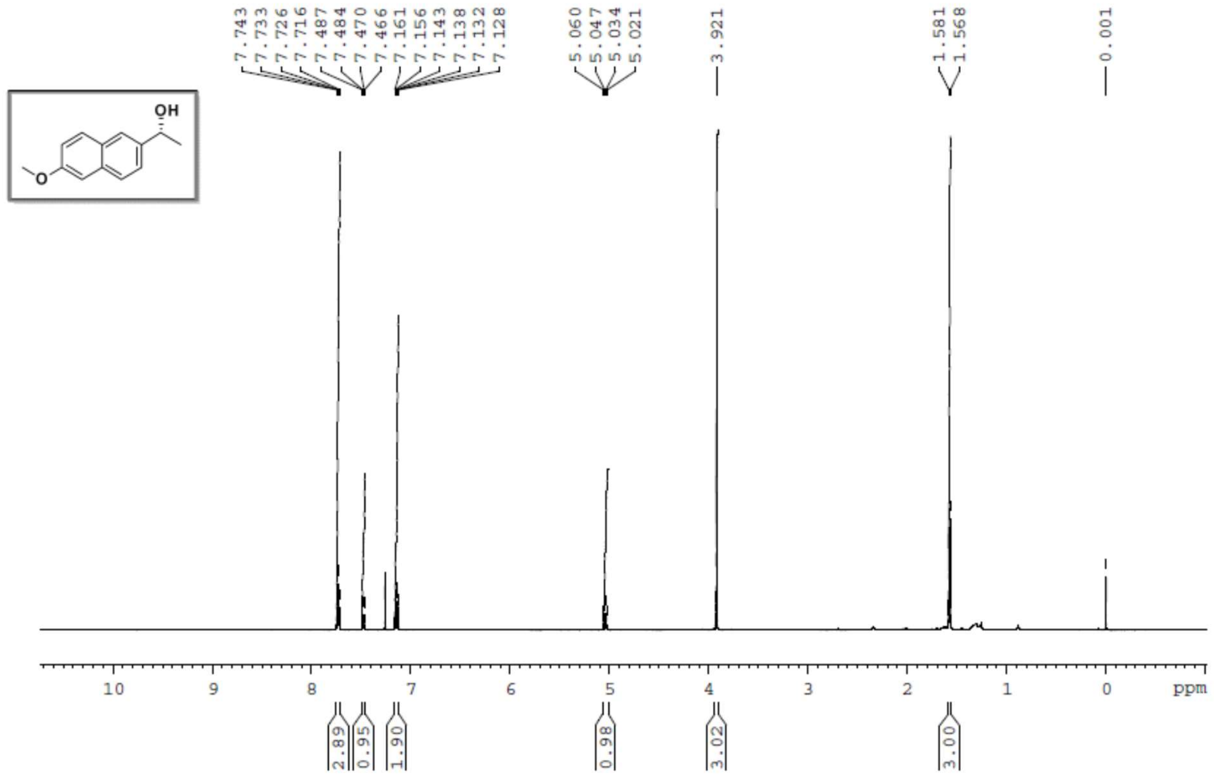

Supplementary Figure 6: ^{1}H NMR spectrum of bio-(*R*)- 1-(6-Methoxynaphthalen-2-yl)ethanol 2b'

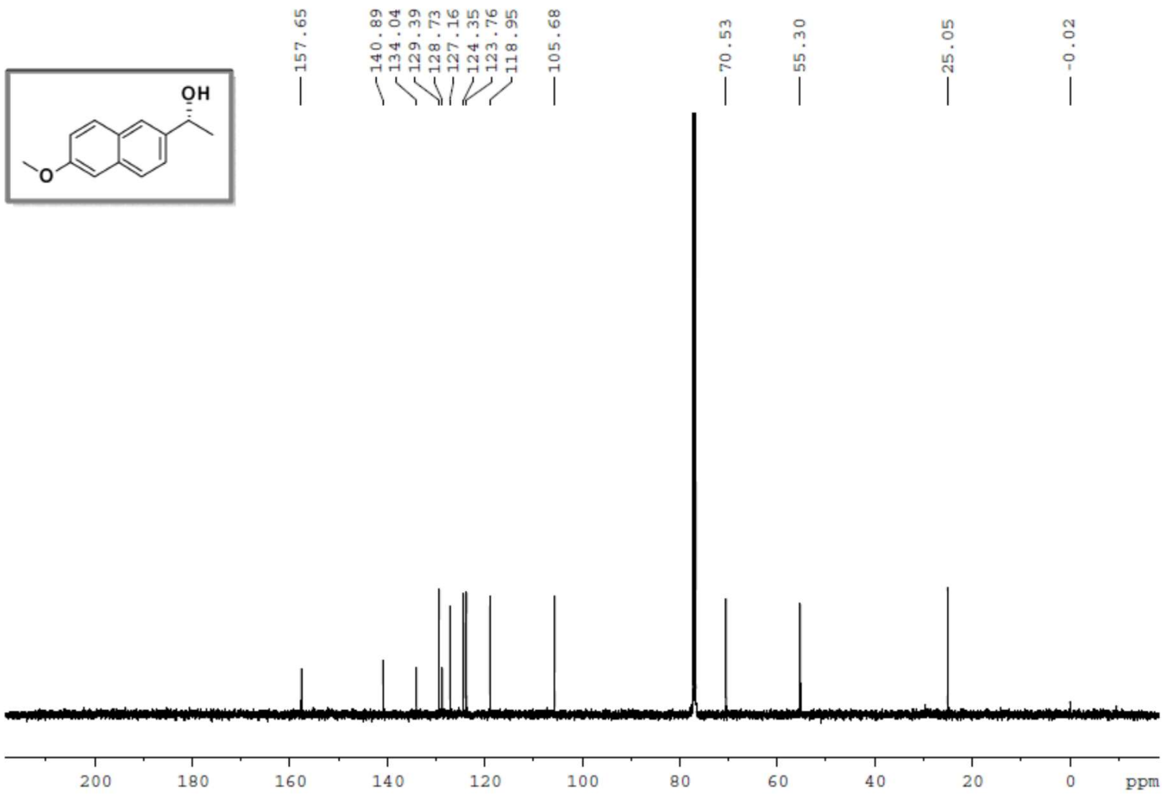

Supplementary Figure 7: ^{13}C NMR spectrum of bio-(*R*)- 1-(6-Methoxynaphthalen-2-yl)ethanol 2b'

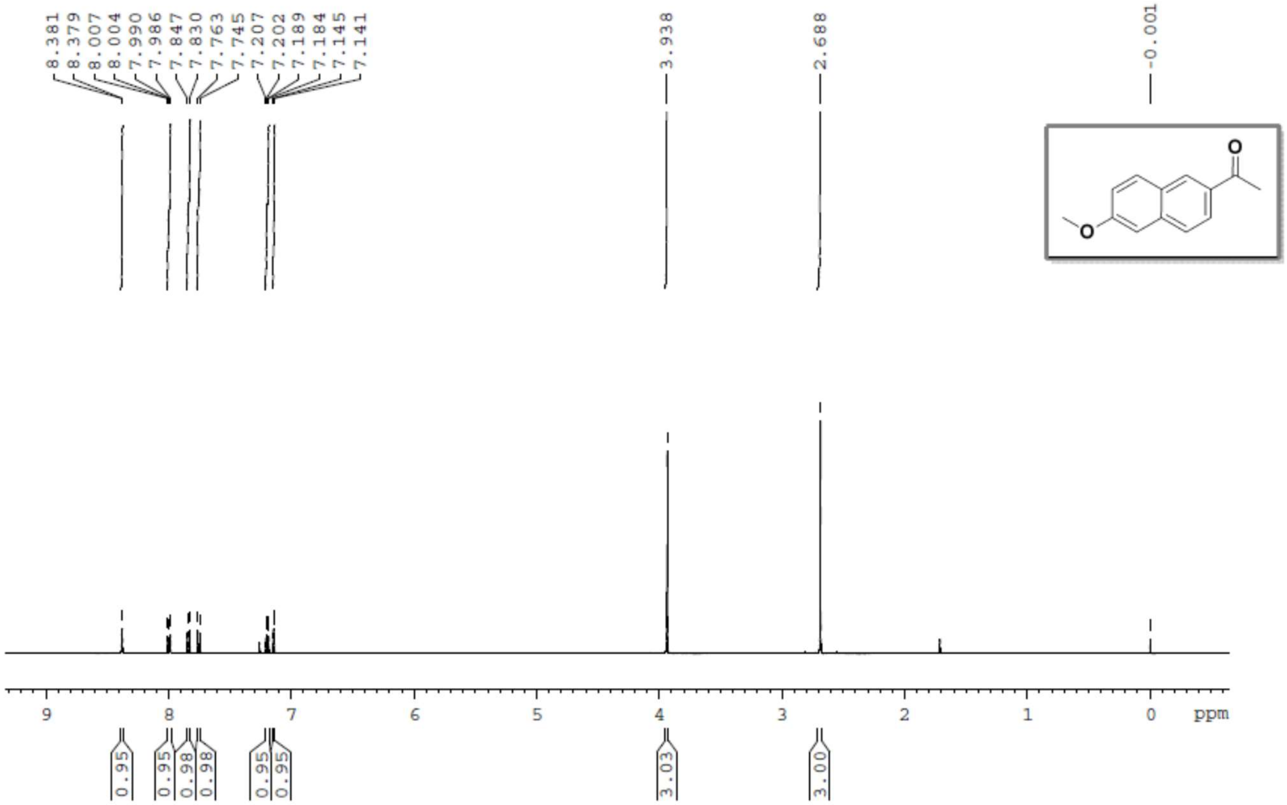

Supplementary Figure 8: ^{1}H NMR spectrum of bio-1-(6-Methoxynaphthalen-2-yl)ethanone 1b

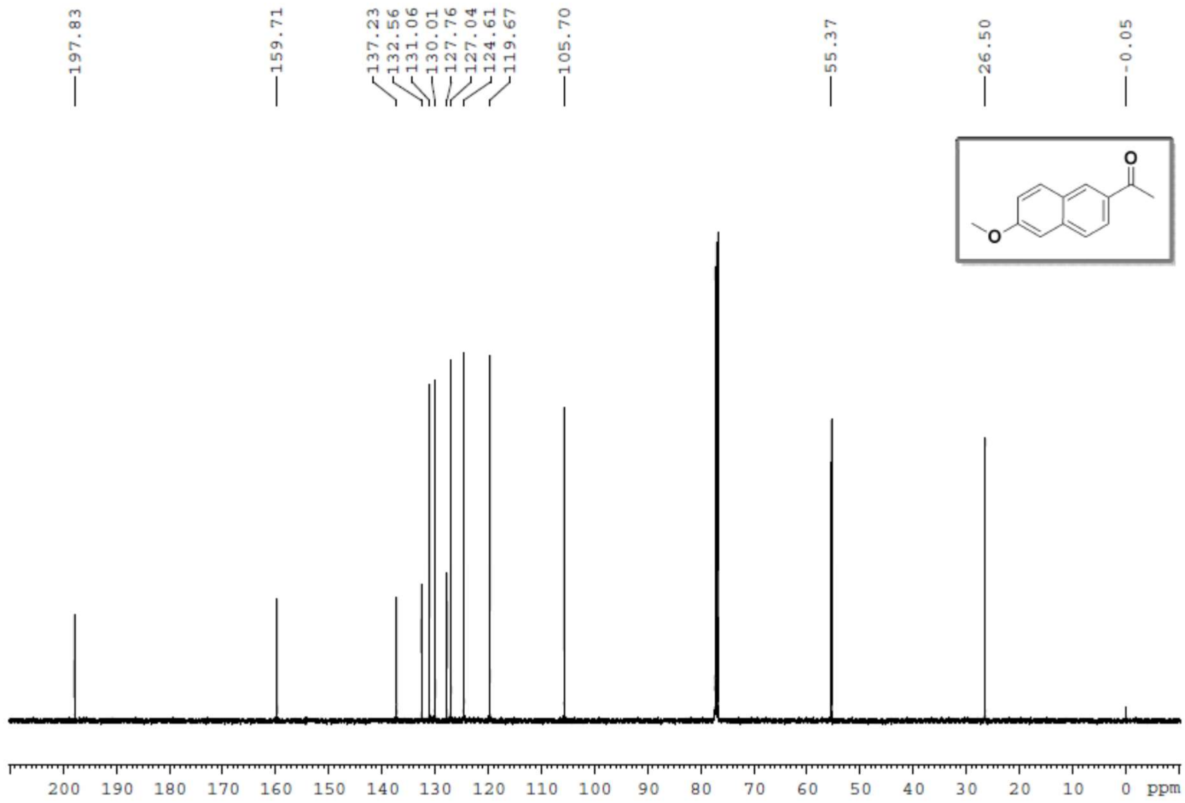

Supplementary Figure 9: ^{13}C NMR spectrum of bio-1-(6-Methoxynaphthalen-2-yl)ethanone 1b

Supplementary Table 1: Instrument settings used for confocal laser microscopy

Parameters	Instrument settings
Dimension	x: 512, y: 512, channels: 2, 8-bit
Image size	x: 127.78 μm, y: 127.78 μm
Scan mode	plane
Zoom	0.5-1.0
Objective	Plan-Apochromat 100x/1.40 Oil DIC M27
Pixel Dwell	3.15 μs
Average	1
Master Gain	Ch1 : 676; ChD : 537
Digital Gain	1.00
Digital offset	Ch1 : 0.00; ChD : 2.00
Pinhole	Ch1 : 84 μm
Filters	Ch1 : 415 - 735
Beam splitters	MBS : MBS 405/488/555/639
	DBS1 : 480 nm
Lasers	405 nm : 11.0 %
